# Metabolomics Approach for Discrimination and Quality Control of Natural and Commercial *Fallopia multiﬂora* Products in Vietnam

**DOI:** 10.1155/2020/8873614

**Published:** 2020-11-06

**Authors:** Nguyen Thi Thoa, Nguyen Hai Dang, Do Hoang Giang, Nguyen Thi Thu Minh, Nguyen Tien Dat

**Affiliations:** ^1^Graduate University of Science and Technology, Vietnam Academy of Science and Technology, 18-Hoang Quoc Viet, Cau Giay, Hanoi, Vietnam; ^2^Faculty of Chemical Technology, Hanoi University of Industry, 298-Cau Dien, Bac Tu Liem, Hanoi, Vietnam; ^3^University of Science and Technology of Hanoi, Vietnam Academy of Science and Technology, 18-Hoang Quoc Viet, Cau Giay, Hanoi, Vietnam; ^4^Center for Research and Technology Transfer, Vietnam Academy of Science and Technology, 18-Hoang Quoc Viet, Cau Giay, Hanoi, Vietnam

## Abstract

A precise HPLC-DAD-based quantification together with the metabolomics statistical method was developed to distinguish and control the quality of *Fallopia multiflora*, a popular medicinal material in Vietnam. Multivariate statistical methods such as hierarchical clustering analysis and principal component analysis were utilized to compare and discriminate six natural and twelve commercial samples. 2,3,4′,5-Tetrahydroxystilbene 2-*O*-*β*-D-glucopyranoside (THSG) **(1)**, emodin **(4)**, and the new compound 6-hydroxymusizin 8-*O*-*α*-D-apiofuranosyl-(1⟶6)-*β*-D-glucopyranoside **(5)** could be considered as important markers for classification of *F. multiﬂora.* Furthermore, seven phenolics were quantified that the variation in the contents of selected metabolites revealed the differences in the quality of natural and commercial samples. Recovery of the compounds from the analytes was more than 98%, while the limits of detection (LOD) and the limits of quantitation (LOQ) ranged from 0.5 to 6.6 *μ*g/ml and 1.5 to 19.8 *μ*g/ml, respectively. The linearity, LOD, LOQ, precision, and accuracy satisfied the criteria FDA guidance on bioanalytical methods. Overall, this method is a promising tool for discrimination and quality assurance of *F. multiflora* products.

## 1. Introduction

*Fallopia multiflora* (Thunberg) Haraldson, synonym Polygonum multiflorum Thunb., is a well-known traditional medicine in several South Asian countries. The roots of this plant have been used to promote hair growth and enhance liver and kidney functions [1]. Previous studies reported that the major compounds, i.e., 2,3,4′,5-tetrahydroxystilbene 2-*O*-*β*-D-glucopyranoside (THSG) and emodin may exhibit hepatoprotective [2–4], antiaging [5, 6], and antioxidant activities [7]. THSG and other stilbenes also showed the antihyperlipidemia effect [8], whereas emodin exhibited significant anticancer, anti-inflammatory, and tonic tension inhibiting effects [9, 10]. Also, polyphenolics, flavonoids, glycosides, tannins, steroids, and other types of compounds were isolated from *F. multiflora* which have shown remarkable antioxidant, immunomodulating, and cerebrovascular protective effects [9–14]. Commonly, roots of *F. multiflora* are processed by steaming with black beans, thus, declining more than half of THSG content [15]. Recent studies showed that the contents of THSG, emodin, emodin-8-*O*-glucoside, and gallic acid could be decreased sharply at high temperature, hence, decreased its antioxidant activity [16, 17]. The phytochemicals of *F. multiflora* may vary due to geographical origins. Several recent studies demonstrated that the contents of THSG, emodin, and physcion might be dramatically different due to the origins of the plants [18–20]. HPLC-fingerprint chromatography and LC-MS based metabolomics approaches are the common methods for geographical and processing discrimination of *F. multiflora,* which illustrated clearly the markers to distinguish the samples with high accuracy [21, 22].

**Figure 1:**
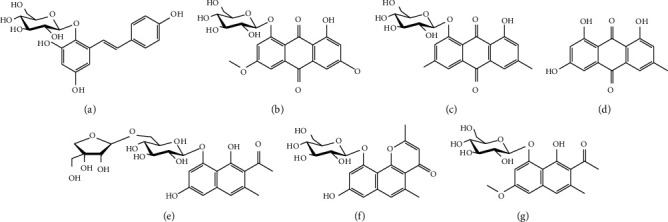
Chemical structures of seven standard compounds. (a) 2,3,4′,5-tetrahydroxystilbene 2-*O*-*β*-D-glucopyranoside. (b) Physcionin. (c) Emodin 8-glucoside. (d) Emodin. (e) 6-hydroxymusizin 8-*O*-*α*-D-apiofuranapiofuranosyl-(1-->6)-*β*-D-glucopyranoside. (f) Pleuropyrone A. (g) Torachrysone 8-*O*-*β*-D-glucopyranoside.

In Vietnam, *F. multiflora* has been widely used in raw materials or processed products [23]. However, there is no effective way to classify, identify, and control the quality of commercial *F. multiflora* products in Vietnam. Recently, we reported about the phenolic constituents from *F. multiflora* roots, including THSG (**1**), physcionin (**2**), emodin 8-glucoside (**3**), and emodin **(4),** pleuropyrone A (**6**), torachrysone 8-*O*-*β*-D-glucopyranoside (**7**), and the naptholic derivative 6-hydroxymusizin 8-*O*-*α*-D-apiofuranosyl-(1⟶6)-*β*-D-glucopyranoside (**5**) (Figure 1) [24]. In the present study, we performed HPLC analyses on the phenolic contents and applied the statistical methods to discriminate the natural roots and commercial medicinal slices of *F. multiflora*. In addition, the contents of these compounds were quantified and compared among the samples.

## 2. Materials and Methods

### 2.1. Sample Preparation

Six samples of 3- to 4-year-old natural roots of *F. multiﬂora*, coded Nat_1 to Nat_6, were collected in six districts of Ha Giang, a northern mountainous province in Vietnam, in October 2015. These samples were identiﬁed by Dr. Nguyen The Cuong, the Institute of Ecology and Biological Resources, Vietnam Academy of Science and Technology. Commercial medicinal slices (CMS) of *F. multiﬂora,* which coded CMS_1 to CMS_12 were purchased at oriental medicine stores in Hanoi, Vietnam, in November 2015. After being cut and powdered, 5 g of each sample was extracted three times in methanol under sonication condition at 30°C. Then, all of the filtrates were concentrated and made up the volume with methanol to 20 ml. The samples could be diluted if necessary. All solutions had been filtered through 0.45 *μ*m Agilent Econoﬁlters (Agilent Technologies, USA) before being injected to the HPLC system. Each sample was analyzed in triplicate.

### 2.2. Prepare Calibration Standards

Seven standard compounds (Figure 1) were isolated from *F. multiﬂora* as described in the previous paper [24]. Purities of these compounds were assessed at more than 95% by HPLC analyses (data not shown). Calibration solutions for each standard compound were prepared at the concentration range of 5–1000 *μ*g/ml. Quality control samples for the compounds were prepared at 25 *μ*g/ml, 125 *μ*g/ml, and 500 *μ*g/ml.

### 2.3. Instrumentation

HPLC-grade solvents were obtained from Scharlau (Spain) and Merck Millipore (Darmstadt, Germany). HPLC-DAD data were obtained on an Agilent Series 1260 (Agilent Technologies, USA) system, consisting of a vacuum degasser, a quaternary mixing pump, an autosampler, a column oven, and a diode-array detector (DAD) using a ZORBAX Eclipse XDB C18 column (150 mm x 4,6 mm, 5 *μ*m) equipped with a ZORBAX Eclipse XDB C18 guard column (12.5 mm × 4.6 mm, 5 *μ*m) (Agilent Technologies, USA). A mixture of 0.1% v/v of acetic acid in water (mobile phase A) and methanol (MeOH) (mobile phase B) was utilized as the mobile phase.

### 2.4. Linearity, LOD, and LOQ

Linearity was evaluated in this concentration range three separate times. The calibration curve obtained had to have a correlation coefficient (R2) at least 0.999 for every analyte. The limit of detection (LOD) was the lowest concentration of the standard compound possibly identified. Meanwhile, the limit of quantitation (LOQ) illustrated the lowest concentration of analyte possibly quantified. LOD and LOQ were obtained using the calibration curve, based on the standard deviation (SD) of the response and the slope of the calibration curve, according to the equations as follows:

(1) $$\begin{array}{c} \text{LOD }=\;3\text{.}3\frac{\sigma }{\text{a}},\\ \text{LOQ }=\;10\frac{\sigma }{\text{a}}, \end{array}$$

where *σ* is the standard derivation of the data response, and a is the slope of the calibration curve.

### 2.5. Precision and Accuracy

The intraday of variation was evaluated by analyzing the response of six replicates of the low, medium, and high concentrations of quality control samples at 25, 125, and 500 *μ*g/ml, respectively, on the same day, whereas the interday variation was evaluated with those quality control samples injected into the HPLC over three consecutive days. The precision of the measurement was assessed by the coefficient of variation (CV%), which was calculated by the ratio of standard deviations of replicates to the mean concentrations. The accuracy was calculated from the recovery of the standard spiking into a sample that had known the contents of the compounds. The mixture was extracted and analyzed using the method mentioned above. Six replicates were performed for the test.

### 2.6. Data Processing and Statistical Analysis

Data acquisition and processing were performed by Agilent ChemStation (Agilent Technologies, USA) and Microsoft Excel 2016 (Microsoft Corporation, USA). The principal component analysis (PCA) and hierarchical clustering analysis (HCA) were performed on the STATISTICA 12 (Dell Software, United States) and R package (R Foundation for Statistical Computing, Vienna, Austria), respectively.

## 3. Results and Discussion

### 3.1. Optimization of HPLC-DAD Conditions

**Figure 2:**
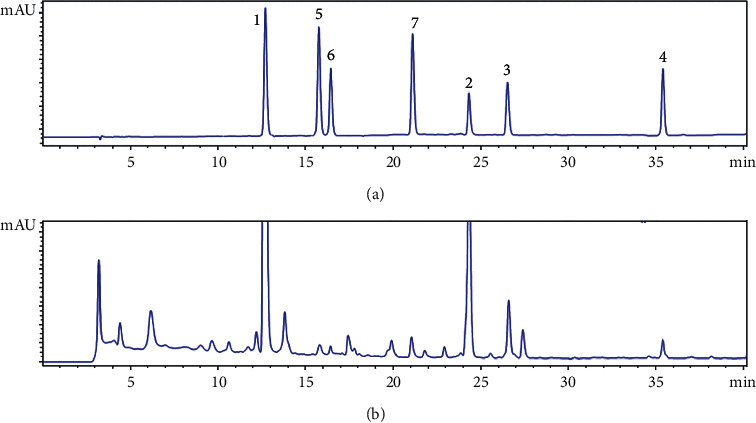
The representative chromatograms at UV 280 nm of (a) a standards mixture and (b) the sample Nat_01. The numbers in brackets indicate seven standard compounds as mentioned above.

An optimized strategy of the HPLC-DAD system was carried out to obtain the separated peaks of the compounds on the chromatogram (Figure 2). All analytes were analyzed on the Agilent Series 1260 system using a ZORBAX Eclipse XDB C18 column (150 mm x 4,6 mm, 5 *μ*m) equipped with a ZORBAX Eclipse XDB C18 guard column (12.5 mm × 4.6 mm, 5 *μ*m) kept at 30°C. A mixture of 0.1% v/v of acetic acid in water and methanol with a linear gradient exhibited a better separation and peak shapes of all analytes. The flow rate was set at 0.5 ml/min with a linear gradient from 30 to 100% methanol over 40 min, followed by 5 min washing with 100% methanol and 5 min of the initial condition. The injection volume for each sample was 5 *μ*l. The UV detector was simultaneously set at 320 nm (for determination of compounds **(1)**, **(5)**, and **(7)**) and 280 nm (to identify compounds **(2)**, **(3)**, **(4)**, and **(6)**). The retention time (Rt) of each compound is shown in Table 1.

### 3.2. Validation of the Method

The calibration curve of each compound was established from at least six appropriate concentrations in triplicate by plotting the peak areas versus the concentrations. As can be seen in Table 2, all calibration curves showed optimized linearities with the average determination coefficient (*R*^2^) higher than 0.999. LOD values were determined between 0.4 to 6.6 *μ*g/ml illustrating a high sensitivity of the analytical method that was sufficient to determine the contents of *F. multiﬂora* samples.

The results of precision and accuracy were shown in Table 1. The intraday and interday precision (CV%) values ranged from 0.05% to 3.5%, whereas the accuracy values calculated from recovery distributed between 98% and 103%. These results showed acceptable precision and accuracy which were complying with the FDA guidance on bioanalytical methods [25]. Therefore, the established method is suitable for the simultaneous quantitation of selected compounds in *F. multiﬂora.*

### 3.3. Metabolomics and Quantification of *F. Multiﬂora* Samples

**Figure 3:**
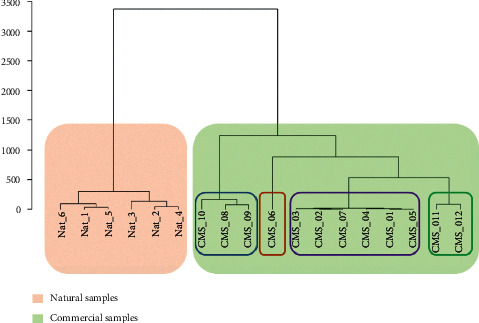
HCA dendrogram of natural and commercial *F. multiﬂora* samples with detected metabolites.

Metabolomics was utilized to identify the similarity and dissimilarity among a large number of samples. In this study, peak areas of seven compounds in 18 samples were collected from the chromatograms at the selected wavelengths and transferred to the tablet form to conduct hierarchical cluster analysis (HCA) and principal component analysis (PCA) for the discrimination of natural and commercial sliced *F. multiﬂora.* HCA was performed based on differences in contents of seven compounds which were represented by peak area values. The Euclidean distances among 18 samples were calculated on R package software that was illustrated on the HCA dendrogram (Figure 3). As can be seen, the group of six natural samples showed a dramatic difference to the commercial sliced samples on the HCA dendrogram. Meanwhile, the commercial medicinal sliced samples clearly clustered among the samples CMS_06, CMS_08 to CMS_10, CMS_11 to CMS_12, and the rest of the CMS samples.

**Figure 4:**
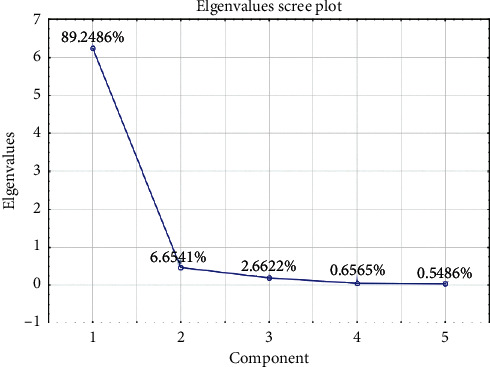
PCA scree plot.

**Figure 5:**
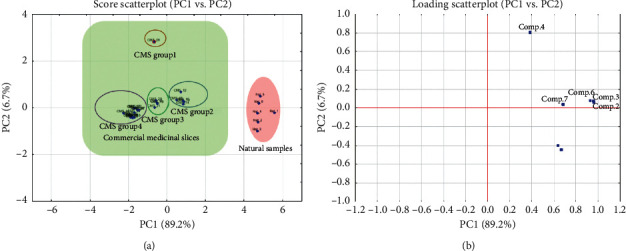
PCA 2D score plot (a) and loading plot (b) of natural and commercial *F* multiflora samples with detected metabolites.

On the other hand, the PCA score plot (Figure 4(a)) was also sharply divided into two regions represented natural samples and other commercial samples, whereas the CMS samples were also separated into four clusters on the PCA plot, which were similar on the HCA dendrogram. As can be seen from the scree plot (Figure 5), the first two principal components (PCs) accounted for more than 90% of the total variation of samples. Thus, we suppose that these PCs carried major information of variables. Considerably, CMS_06 was determined as a specific sample on the PCA plot. These results indicated a clear difference in the contents of standard compounds between natural and commercial products of *F. multiﬂora.* Furthermore, CMS samples could be categorized into four CMS groups: group 1: CMS_06; group 2: CMS_08 to CMS_10; group 3: CMS_11 to CMS_12; group 4: rest of CMS samples, based on the results of PCA and HCA. Marker compounds for the clustering could be identified on the basis of the PCA loading plot (Figure 4(b)). As can be seen, emodin **(4)**, THSG **(1)**, and 6-hydroxymusizin 8-*O*-*α*-D-apiofuranosyl-(1⟶6)-*β*-D-glucopyranoside **(5)** could be clearly detected as marker compounds to distinguish *F. multiﬂora* samples.

For a further explanation of the discrimination among the samples, contents of seven selected standards were quantified using the method above. The results were calculated and summarized in Table 3.

The contents of each compound varied considerably in different *F. multiﬂora* samples. THSG **(1)** contents in natural samples ranged from 26,211 to 55,010 mg/kg, which were 4 to 108-fold higher than in CMS samples. THSG contents also varied widely among CMS groups as categorized above. CMS group 2 contained the highest contents of THSG among four CMS groups at more than 9,000 mg/kg, followed by CMS group 3 at about 5,000 mg/kg, whereas THSG contents of the sample CMS_01 to CMS_07 (CMS groups 1 and 4) varied at low levels, from 507 to 3,285 mg/kg. Previous studies also reported that THSG contents may range from 8,000 to 60,000 mg/kg of natural *F. multiﬂora*, and at sharply lower levels in commercial samples [16, 18, 21]. Contents of the compound, 6-hydroxymusizin 8-*O*-*α*-D-apiofuranosyl-(1⟶6)-*β*-D-glucopyranoside **(5)**, in natural samples were dramatically higher than other CMS samples, while CMS group 2 showed the highest content of the compound among four CMS groups. Considerably, very low or no contents of physcionin **(2),** emodin 8-glucoside **(3)**, pleuropyrone A **(6)**, and torachrysone 8-*O*-*β*-D-glucopyranoside **(7)** were determined in samples of CMS group 1 and 4, while natural samples showed the highest contents of these metabolites, which were at least 3-fold higher than the values of CMS groups 2 and 3. Interestingly, emodin **(4)** was found at the highest level in the sample CMS_06 (CMS group 1), followed by natural samples, CMS group 2, and CMS group 3, respectively, while detected at low contents in CMS group 4. This compound might be useful as a marker that led to the clear separation of the sample CMS_06 to the other analytes on the PCA score plot. A study on processing *F. multiﬂora* illustrated that the emodin content may increase 20–30% after steaming with or without black beans for a long time [15]. Thus, CMS_06 might be processed in a related condition which sharply increased the synthesis of emodin.

Overall, except emodin, six other standards showed higher contents in natural samples that might lead to discrimination to CMS samples. The clusters of samples in CMS groups could base on the different contents of THSG **(1)**, physcionin **(2)**, emodin 8-glucoside **(3)**, 6-hydroxymusizin 8-*O*-*α*-D-apiofuranosyl-(1⟶6)-*β*-D-glucopyranoside **(5)**, pleuropyrone A **(6)**, and torachrysone 8-*O-β*-D-glucopyranoside **(7)**, where CMS group 2 contained higher contents of standards than CMS groups 3 and 4, respectively. CMS group 1, included only the sample CMS_06, was classified by a significantly high content of emodin **(4)**. Considerably, the variation in contents of 6-hydroxymusizin 8-*O*-*α*-D-apiofuranosyl-(1⟶6)-*β*-D-glucopyranoside **(5)** among analytes showed a similar trend as THSG (**1**), thus, could be used as a new marker for classification of *F. multiﬂora*. In general, commercial *F. multiﬂora* products were processed by steaming with black bean or stored under unappropriated conditions, which notably decreased the contents of metabolites [9]. The previous study reported that and 50% of stilbenes contents might decompose after high-temperature processing, which reduced sharply the toxicity of *F. multiﬂora* [15]. However, THSG and emodin were proved as the major anticancer, antioxidant, and antivirus agents of *F. multiﬂora* [25–36]*. T*hus, the decrease in these compounds may lead to a serious decline in the quality of medicine. In addition, the obtained results showed a variation of THSG contents in CMS samples, in which the contents of THSG in natural samples may be up to 108-fold higher than CMS samples. A previous study on *F. multiﬂora* also illustrated a high difference in the contents of major metabolites between natural and commercial processed products. Accordingly, THSG or emodin contents of natural roots might be up to 130-fold higher than in commercial products [22]. The variation of compositions of CMS samples in this study indicated that the quality of commercial *F. multiﬂora* may vary significantly among manufactures due to different processing or preserving methods. Therefore, further studies should be carried out to explain clearly the reason and consequences of these issues.

## 4. Conclusions

For the first time, an HPLC-DAD based metabolomics approach was performed to discriminate the natural and commercial medicinal slices of *F. multiﬂora* roots in Vietnam. Moreover, seven compositions were quantified to evaluate the quality of natural and commercial samples. With this approach, the natural *F. multiﬂora* samples were significantly distinguished from the commercial ones. Large different contents of seven selected metabolites among two categories of samples indicated dramatic changes of *F. multiﬂora* chemical compositions on processing and manufacturing. On the other hand, the discrimination based on the difference of standard metabolites of commercial samples performed a considerable variation of *F. multiﬂora* quality among manufactures. Because *F. multiﬂora* is a common traditional medicine, its quality needs to be strictly controlled. Besides emodin and THSG, interestingly, the new compound, 6-hydroxymusizin 8-*O*-*α*-D-apiofuranosyl-(1⟶6)-*β*-D-glucopyranoside was identified as a marker for the discrimination. Overall, this study presented an effective method for precisely distinguishing natural and commercial *F. multiﬂora* products, also possibly applying for quality evaluation of other diverse medicinal herbs.

## Figures and Tables

**Table 1:** Intra- and Interday precision and accuracy of seven standard compounds in *F. multiﬂora.*

Analyte	Rt (min)	C (*μ*g/ml)	Intraday (*n* = 6)		Interday (*n* = 6)	
			Precision (CV%)	Accuracy (% from recovery) mean ± SD	Precision (CV%)	Accuracy (% from recovery) mean ± SD
**(1)**	12.7	25	0.7736	100.19 ± 4.98	1.7772	98.68 ± 8.35
		125	0.1587	98.68 ± 2.36	0.6515	100.64 ± 2.17
		500	0.9496	99.49 ± 0.82	0.8775	99.61 ± 1.95

**(2)**	24.3	25	1.7564	99.94 ± 4.82	1.6769	100.86 ± 5.14
		125	0.1958	99.93 ± 2.04	0.7853	99.82 ± 2.04
		500	0.0788	99.97 ± 0.32	0.1215	100.42 ± 0.57

**(3)**	26.5	25	1.0490	99.38 ± 4.03	1.4874	99.28 ± 3.3
		125	0.5440	98.4 ± 0.99	0.5175	98.9 ± 0.94
		500	0.2066	99.48 ± 0.49	0.2465	98.97 ± 0.54

**(4)**	35.3	25	0.3930	98.37 ± 2.6	0.5160	98.42 ± 5.16
		125	0.0768	98.58 ± 0.91	0.2293	99.27 ± 1.22
		500	0.0671	100.05 ± 0.42	1.4881	100.52 ± 0.64

**(5)**	15.7	25	3.4528	99.64 ± 3.12	3.4721	102.99 ± 6.06
		125	1.2993	100.71 ± 1.05	1.2698	98.1 ± 2.73
		500	0.9496	100.13 ± 0.29	0.7438	103.09 ± 0.54

**(6)**	16.4	25	1.8823	98.23 ± 4.37	1.7781	99.05 ± 4.42
		125	1.2309	100.56 ± 1.27	1.2267	101.02 ± 1.40
		500	0.3624	99.8 ± 0.41	0.3514	99.97 ± 0.43

**(7)**	21.0	25	2.2353	100.45 ± 4.99	2.0892	98.7 ± 8.22
		125	1.3567	99.54 ± 1.78	1.1548	99.54 ± 1.78
		500	0.9318	99.77 ± 0.25	0.7520	98.75 ± 0.80

**Table 2:** Regression data for all standards with LOD and LOQ.

Analyte	Slope^*∗*^	Intercept	*R* ^2^	LOD (*μ*g/ml)	LOQ (*μ*g/ml)
**(1)**	6.1379	3.9659	0.9999	0.5106	1.5474
**(2)**	4.0861	21.5671	0.9998	0.4997	1.5143
**(3)**	2.0617	9.3186	0.9996	0.7349	2.2269
**(4)**	6.6230	−35.4954	0.9998	6.6000	19.8000
**(5)**	1.0218	0.7870	0.9999	1.3600	4.1212
**(6)**	2.3317	6.8226	0.9999	1.4333	4.3435
**(7)**	0.9279	2.4082	0.9998	1.5736	4.7684

^*∗*^Experiments were performed in triplicate.

**Table 3:** The contents of seven compounds in eighteen *F. multiﬂora* samples.

Group^*∗*^	Sample	Content (mg/kg dry weight)^*∗∗*^						
		(1)	(2)	(3)	(4)	(5)	(6)	(7)
Natural samples	Nat_1	55010 ± 2525	3183 ± 135.5	1404 ± 72.59	438.13 ± 14.09	173.4 ± 9.13	117.0 ± 6.23	398.0 ± 17.03
	Nat_2	46946 ± 3188	3175 ± 234.3	1368 ± 48.97	333.5 ± 14.54	148.7 ± 4.99	100.6 ± 3.34	383.5 ± 9.04
	Nat_3	50491 ± 2826	3170 ± 155.2	1399 ± 86.74	268.3 ± 14.37	170.5 ± 10.84	115.5 ± 7.32	342.1 ± 11.49
	Nat_4	33660 ± 2393	3190 ± 260.3	1405 ± 53.36	354.2 ± 23.93	149.6 ± 7.12	117.3 ± 5.25	411.3 ± 23.68
	Nat_5	29176 ± 1923	3199 ± 216.2	1386 ± 86.71	457.9 ± 14.92	155.7 ± 5.07	131.4 ± 4.63	361.7 ± 22.64
	Nat_6	26211 ± 678.9	3223 ± 192.0	1375 ± 35.47	404.8 ± 26.64	157.8 ± 8.81	114.6 ± 6.50	459.6 ± 21.05

CMS group 1	CMS_06	1698 ± 111.9	+	+	547.0 ± 25.65	35.73 ± 1.30	+	ND

CMS group 4	CMS_01	1170 ± 66.92	ND	+	+	20.41 ± 0.97	+	+
	CMS_02	2160 ± 207.1	+	ND	+	25.64 ± 0.58	+	+
	CMS_03	507.0 ± 23.27	+	+	+	ND	ND	ND
	CMS_04	2285 ± 145.3	+	+	+	43.37 ± 1.55	ND	ND
	CMS_05	3285 ± 282.2	+	+	+	38.81 ± 1.78	+	ND
	CMS_07	2418 ± 110.7	+	+	+	39.40 ± 1.45	ND	ND

CMS group 2	CMS_08	9094 ± 435.1	985.0 ± 45.11	326.6 ± 14.96	93.89 ± 2.97	62.67 ± 2.61	63.57 ± 3.00	219.2 ± 11.33
	CMS_09	11854 ± 965.8	907.2 ± 32.28	351.2 ± 11.52	104.3 ± 3.48	75.11 ± 1.76	71.00 ± 2.01	202.7 ± 6.77
	CMS_10	10470 ± 692.1	818.6 ± 53.75	340.7 ± 24.57	119.0 ± 4.26	90.48 ± 3.93	78.21 ± 3.86	198.3 ± 4.65

CMS group 3	CMS_11	5335 ± 298.2	427.4 ± 22.98	234.3 ± 3.68	112.6 ± 4.89	34.81 ± 1.51	42.87 ± 1.73	139.6 ± 7.46
	CMS_12	4770 ± 328.6	508.9 ± 40.50	203.5 ± 10.29	99.67 ± 1.28	29.81 ± 0.96	52.27 ± 2.05	153.3 ± 6.46

^*∗*^ Categorized by HCA and PCA. ^*∗∗*^Experiments were performed in triplicate. +: Identifiable under the limit of quantitation.; ND : not detected.

## Data Availability

All data supporting the findings of this study are included within the article.
